# Machine learning based multimodel prediction of poor wellbeing, generalized anxiety disorder, and insomnia among adults in Indian urban informal settlements: toward explainable digital mental health screening

**DOI:** 10.3389/fdgth.2026.1935205

**Published:** 2026-09-30

**Authors:** Rupeshkumar Deshmukh, Sudhanshu Mahajan, Reva Wani

**Affiliations:** 1 Department of Community Medicine, Bharati Vidyapeeth (Deemed to be University) Medical College, Pune, India; 2 Medical Intern, Bharati Vidyapeeth (Deemed to be University) Medical College, Pune, India

**Keywords:** anxiety, cluster-aware validation, digital health, explainable artificial intelligence, insomnia, machine learning, mental health, screen time

## Abstract

**Introduction:**

Mental health morbidity among urban informal settlements-dwelling populations is shaped by a complex interplay of behavioural exposures including screen time, working hours, and sleep duration and sociodemographic vulnerability.

**Methods:**

We analyzed cross-sectional data from 1,600 adults recruited from 16 clusters within the urban informal settlement field practice area of a medical college's Urban Health Training Centre in Pune, India, to develop and compare ten supervised machine learning algorithms predicting poor wellbeing (WHO-5), moderate-to-severe generalized anxiety disorder (GAD-7), and moderate-to-severe insomnia (ISI); internal consistency of all three instruments was confirmed from item-level responses. Because participants were nested within 16 clusters, our primary analysis used leave-one-cluster-out (LOCO) cross-validation, in which each cluster was held out in turn as an independent test set; a conventional participant-level 80/20 split with 10-fold cross-validation was retained as a secondary analysis. Model discrimination, calibration, and clinical utility were assessed using AUC, sensitivity, specificity, predictive values, Brier score, decision curve analysis, and DeLong's test, each with cluster-level bootstrap 95% confidence intervals. SHAP was used to interpret the best-performing model, and feature-attribution concordance was additionally assessed across the Rashomon set of near-equivalent top models.

**Results:**

The WHO-5, GAD-7, and ISI showed acceptable-to-excellent internal consistency in this sample (Cronbach's alpha 0.730–0.938; McDonald's omega 0.815–0.953). LightGBM achieved the highest primary (LOCO-CV) AUC for all three outcomes: 0.977 (95% CI 0.970–0.983) for poor wellbeing, 0.987 (95% CI 0.984–0.992) for GAD, and 0.988 (95% CI 0.982–0.993) for insomnia. Several sociodemographic predictors showed unusually strong univariate associations with the outcomes, traced to a repeating between-cluster pattern in the sampling frame; this structure means that even cluster-aware validation may remain optimistic relative to a genuinely external population, a limitation we discuss in detail. SHAP-based rankings were highly concordant (Spearman *ρ* = 0.84–0.95) across the three top-performing, statistically near-equivalent models. A class-weighting sensitivity analysis improved positive predictive value for poor wellbeing from 0.478 to 0.600 with minimal AUC cost.

**Conclusion:**

Gradient-boosted tree ensembles, particularly LightGBM, showed strong and internally consistent discrimination for these three outcomes, assessed using reliable instruments; however, given the between-cluster homogeneity identified in this sample, external validation in an independent population is essential before any claim of generalizable clinical utility.

## 1 Introduction

Common mental disorders including poor psychological wellbeing, generalized anxiety, and insomnia account for a substantial and growing share of the global disease burden (1). These conditions are highly prevalent and frequently co-occurring among adults living in resource-constrained urban informal settlements, where chronic socioeconomic stress, overcrowding, occupational insecurity, and sociodemographic vulnerability converge to elevate psychological morbidity (2). Conventional epidemiological approaches to identifying at-risk individuals typically rely on regression-based association models that assume linear, additive relationships between exposures and outcomes, and that are not optimized for individual-level risk prediction (3). In contrast, machine learning (ML) algorithms can capture complex, non-linear, and interactive relationships among a large number of candidate predictors, and have increasingly been applied to mental health risk prediction across varied populations (4–6).

A persistent barrier to the clinical and public-health adoption of ML models has been their limited interpretability. The emergence of explainable artificial intelligence (XAI) techniques, most notably SHapley Additive exPlanations (SHAP) (7), has addressed this barrier by providing both global and individual-level attribution of each predictor's contribution to model output, grounded in cooperative game theory. A second, related barrier specific to ML applications in clustered or hierarchically sampled health data is the risk that naive, participant-level train/test partitioning overstates real-world generalizability when observations within the same cluster (e.g., the same community or facility) are more similar to one another than to observations from other clusters. We address both barriers directly in this study: we use SHAP for explainability, and we treat cluster-aware validation rather than a conventional random split as our primary analytical design, given that our own sample is nested within 16 geographically defined clusters.

A further, closely related concern is that an explanation derived from a single ‘best’ model may not generalize to the other models that fit the data almost equally well. Breiman first described this phenomenon as the ‘Rashomon effect’: the common empirical observation that many structurally distinct models differing in the variables they rely on, their internal logic, and their case-level predictions can nonetheless achieve statistically indistinguishable predictive accuracy on the same dataset (8). The collection of such near-equally-accurate models is now formally termed the ‘Rashomon set’, and a growing body of work has shown that variable-importance rankings, and by extension SHAP-based explanations, can diverge meaningfully across models within this set even when their discrimination is statistically indistinguishable a phenomenon termed predictive or explanatory multiplicity (9, 10). This multiplicity has been documented across domains including criminal recidivism risk, credit scoring, and healthcare, and has been identified as a source of arbitrary or unjustified conclusions when only a single model from an equally plausible set is reported and interpreted (11, 12). Despite its practical importance for the credibility of explainable-AI-based clinical and public-health prediction models, explicit Rashomon-set analysis remains uncommon in applied mental-health and community-health machine learning research, where a single best-performing model's SHAP output is typically reported without examining whether statistically comparable alternative models would support the same explanatory conclusions (13). This study addresses this gap directly by evaluating the concordance of SHAP-based feature importance across the Rashomon set of top-performing, statistically near-equivalent models identified via DeLong's test, rather than relying on a single best-performing model's explanation alone.

Screen time, working hours, and sleep duration are modifiable behavioural exposures that have each been independently associated with poor mental wellbeing, anxiety, and insomnia. Excessive screen exposure has been consistently linked to depression, anxiety, and lower wellbeing in adults (14–16); short sleep duration and insomnia symptoms are well-established risk factors for depressive and anxiety disorders (17–19); and long working hours (≥55 h/week) have been associated with elevated risk of depressive and anxiety symptoms in multiple prospective cohorts and meta-analyses (20–22). This study aimed to (i) develop and compare ten machine learning algorithms for predicting poor wellbeing, moderate-to-severe GAD, and moderate-to-severe insomnia among adults in an Indian urban informal settlement; (ii) rigorously evaluate model discrimination and classification performance using a cluster-aware primary analysis alongside a conventional participant-level secondary analysis, with bootstrap confidence intervals and formal statistical comparison between models; and (iii) interpret the best-performing model, and the concordance of the near-equivalent Rashomon set of top models, using SHAP (7).

## 2 Materials and methods

### 2.1 Study design and setting

This was a community-based cross-sectional study conducted in the urban informal settlement field practice area of the Urban Health Training Centre (UHTC), Department of Community Medicine, Bharati Vidyapeeth (Deemed to be University) Medical College, Pune, Maharashtra, India, between May 2022 and December 2022. The UHTC field practice area comprises 33 Anganwadi (government-run community childcare and nutrition centre) catchment zones, which served as the natural community-level sampling units for this study. A structured, interviewer-administered questionnaire was used to collect sociodemographic, behavioural, and mental health outcome data. The study adhered to the principles of the Declaration of Helsinki, and ethical approval was obtained from the Institutional Ethics Committee of Bharati Vidyapeeth (Deemed to be University) Medical College, Pune (approval number BVDUMC/IEC/05) prior to commencement. All participants were adults (≥18 years) and provided their own written informed consent prior to enrolment; no participant required guardian or next-of-kin consent.

### 2.2 Participants and sample

A two-stage cluster sampling method was used. In the first stage, 16 of the 33 Anganwadi catchment areas within the UHTC field practice area were selected by simple random sampling to serve as the 16 study clusters. In the second stage, 100 households were selected from each of the 16 selected Anganwadi areas by systematic random sampling from the Anganwadi's household roster, yielding a total of 1,600 households. From each selected household, one eligible adult respondent was chosen using the Kish grid method, a standardized table-based technique for randomly selecting a single respondent from among all eligible household members, minimizing selection bias from interviewers preferentially approaching the most readily available household member. All adults aged 18 years or older who had resided in the informal settlement for at least 6 months were eligible. Individuals who did not provide consent, households where the door was locked during the survey period, and persons with a pre-existing mental disorder were excluded. This procedure yielded a final analytic sample of 1,600 adult participants.

### 2.3 Outcome measures

Three binary outcomes were defined, each dichotomized from a validated instrument using a cut-off fixed *a priori* from the published literature.

Outcome definitions: The WHO-5 Well-Being Index consists of five items scored from 0 to 5, yielding a raw score of 0–25, which is commonly transformed to a 0–100 scale; higher scores indicate better well-being, with a standardized score <50 (raw score 0–12) indicating poor well-being. The Generalized Anxiety Disorder-7 (GAD-7) scale ranges from 0 to 21, with scores of 0–4, 5–9, 10–14, and 15–21 representing minimal, mild, moderate, and severe anxiety symptoms, respectively; in the present study, moderate-to-severe anxiety was defined as a GAD-7 score of ≥10. The Insomnia Severity Index (ISI) ranges from 0 to 28, with scores of 0–7, 8–14, 15–21, and 22–28 indicating no clinically significant insomnia, subthreshold insomnia, moderate clinical insomnia, and severe clinical insomnia, respectively; in the present study, moderate-to-severe insomnia was defined as an ISI score of ≥15. Thus, the primary binary outcomes for the analysis were poor well-being (WHO-5 < 50), moderate-to-severe anxiety symptoms (GAD-7 ≥ 10), and moderate-to-severe insomnia (ISI ≥15).

Applying these definitions to the study sample: poor wellbeing (WHO-5 < 50) was present in 219/1,600 participants (13.7%) (23); moderate-to-severe GAD (GAD-7 ≥ 10) in 285/1,600 (17.8%) (24); and moderate-to-severe insomnia (ISI ≥15) in 329/1,600 (20.6%) (25). A binary framing was chosen *a priori*, rather than an ordinal or multi-class framing, because the motivating clinical/public-health question was individual-level identification of cases warranting further assessment, a binary screening decision consistent with how these instruments are used operationally in primary care and community screening. Dichotomization at the pre-specified cut-offs reproduced the binary categories used throughout this manuscript exactly for all 1,600 participants (100% agreement) when independently re-derived from the total/standardized scores, confirming the outcome variables were applied consistently. The full distribution across the ordinal severity bands for GAD-7 and ISI, descriptive statistics for all three total/standardized scores, and the internal-consistency reliability of each instrument in this sample are reported in Tables 1, 2.

**Table 1:** Baseline sociodemographic and behavioural characteristics of the study sample (*N* = 1,600), including categorized primary behavioural exposures (screen time, working hours, and sleep hours) and outcome prevalence.

Variables	Category	Frequency (n)	Percentages (%)
**Gender**	Male	856	53.5%
	Female	744	46.5%
**Age Group (years)**	Less than equal to 40	801	50.1%
	>40	799	49.9%
**Family Type**	Joint	871	54.4%
	Nuclear	729	45.6%
**Religion**	Hindu	1233	77.1%
	Other	367	22.9%
**Marital Status**	Married ever	1526	95.4%
	Unmarried	74	4.6%
**Education**	Less than equal to SSC	858	53.6%
	>SSC	742	46.4%
**Occupation**	Skilled	970	60.6%
	Unskilled	630	39.4%
**Socioeconomic Status**	SES(IV + V)	894	55.9%
	SES(I + II + III)	706	44.1%
**House Ownership**	No	841	52.6%
	Yes	759	47.4%
**Debt/Loan**	No	1053	65.8%
	Yes	547	34.2%
**Substance Abuse**	Yes	1237	77.3%
	No	363	22.7%
**Physical Activity**	No	1181	73.8%
	Yes	419	26.2%
**Family History of Mental Illness**	No	1392	87%
	Yes	208	13%
**Chronic Illness History**	No	1348	84.2%
	Yes	252	15.8%
**Social Group Participation**	Present	963	60.2%
	Absent	637	39.8%
**Screen Time**	<4 h/day	745	46.6%
	4–6 h/day	774	48.4%
	>6 h/day	81	5.1%
**Working Hours**	≤8 h/day	743	46.4%
	>8–10 h/day	763	47.7%
	>10 h/day	94	5.9%
**Sleep Hours**	<6 h/day	292	18.2%
	6–8 h/day	1092	68.2%
	>8 h/day	216	13.5%
**Mental Health Outcomes**	Poor Wellbeing	219	13.7%
	Moderate to Severe GAD	285	17.8%
	Moderate to Severe Insomnia	329	20.6%

**Table 2:** Internal consistency reliability of the WHO-5, GAD-7, and ISI in the study sample, computed from item-level responses (*N* = 1,600).

Instrument	N items	N participants	Cronbach's alpha	McDonald's omega	Mean inter-item r	First-factor variance explained
WHO-5	5	1600	0.938	0.953	0.754	80.4%
GAD-7	7	1600	0.73	0.815	0.284	39.4%
ISI	7	1600	0.909	0.934	0.614	67.2%

### 2.4 Candidate predictors and data quality

Eighteen candidate predictors were considered for each outcome. Four were recorded as continuous values at the point of data collection: age (years), screen time (hours/day), working hours (hours/day), and sleep hours (hours/day). Categorization of these four variables was applied afterward, as a data-processing step subsequent to collection, rather than reflecting how the data were originally gathered: age was dichotomized (≤40 vs. > 40 years) for both descriptive reporting and as a model input, following standard epidemiological convention for this population, while screen time, working hours, and sleep hours were categorized into bands only for descriptive reporting in Table 1 and were retained in their original continuous form as inputs to the machine learning models. The remaining fourteen predictors were binary-coded sociodemographic and behavioural covariates collected as categorical variables from the point of data collection: gender, family type (nuclear vs. joint), religion, marital status, education (≤SSC vs. >SSC), occupation (skilled vs. unskilled), socioeconomic status, house ownership, presence of debt/loan, substance abuse, physical activity, family history of mental illness, chronic illness history, and social group participation.

Structured questionnaires were administered face-to-face by trained interviewers. The fourteen categorical covariates were collected using closed-ended response items: interviewers read out a fixed set of response options (e.g., Yes/No; a labelled education/occupation band) and recorded the participant's selected category directly, rather than transcribing open-ended free-text responses. The four continuous variables (age, screen time, working hours, sleep hours) were each entered into a structured field with a plausible range restriction (e.g., 0–24 h/day for the three behavioural exposures). The three outcome instruments (WHO-5, GAD-7, ISI) were administered and scored as continuous/ordinal totals from their respective item sets and subsequently dichotomized using the standard cut-offs described above. The predominantly closed-category, interviewer-administered format for the categorical covariates, together with structured, range-restricted numeric fields for the continuous variables, is the primary reason 100% completeness was achievable: every item required the interviewer to actively record a valid response a selected category or an in-range numeric value before the form could be considered complete, which is a materially different completion mechanism from a self-administered or open-ended survey instrument, where item non-response is far more common. Each form was checked for completeness by the interviewer before leaving the household and re-checked by a field supervisor before data entry. There were no missing values for any of the 23 study variables across all 1,600 participants; no imputation was performed at any stage, and the complete-case dataset was used for all analyses.

Outcome prevalence and several predictor distributions were examined for homogeneity across the 16 sampling clusters. Rather than varying smoothly and independently, they recur in a pattern of distinct statistical profiles across the 16 clusters, outcome prevalence and predictor distributions with one another (full cluster-level statistics are provided in Supplementary Table S1). This pattern bears directly on how the primary and secondary validation analyses should be interpreted.

### 2.5 Machine learning pipeline, cluster-aware primary analysis, and preprocessing/leakage control

Ten supervised classification algorithms, spanning linear, tree-based, ensemble, kernel, instance-based, and neural network paradigms, were trained for each outcome: logistic regression (3), decision tree, random forest (26), gradient boosting machine (27), AdaBoost, extreme gradient boosting (XGBoost) (28), light gradient boosting machine (LightGBM) (29), support vector machine with radial basis function kernel, k-nearest neighbours (k = 15), and a multilayer perceptron neural network. Continuous predictors were standardized (zero mean, unit variance) and categorical predictors were binary-coded; standardization was implemented inside a single scikit-learn Pipeline object together with each classifier, so that the scaler was always re-fit on the training partition only within each cross-validation fold, never on pooled data.

Primary analysis- cluster-aware leave-one-cluster-out (LOCO) cross-validation: because participants are nested within 16 geographically defined clusters, our primary validation design used leave-one-cluster-out cross-validation. For each of the 16 clusters in turn, all ten models were trained on the remaining 15 clusters (1,500 participants) and evaluated on the fully held-out cluster (100 participants); out-of-fold predicted probabilities were pooled across all 16 folds to yield one cluster-independent prediction for every one of the 1,600 participants. The classification threshold for each model was derived from these pooled out-of-fold predictions using the Youden index (maximizing sensitivity + specificity−1).

Secondary analysis participant-level split: for comparison with more conventional reporting in this literature, we retained a secondary analysis using a random 80%/20% participant-level split (stratified by outcome), with model training and threshold selection via stratified 10-fold cross-validation on the 80% training partition and one-time evaluation on the untouched 20% test partition, exactly as in our original submission. This analysis is reported in Table 4 and is explicitly secondary; it is not used to support the study's headline performance claims.

A fixed random seed (42) was used throughout for reproducibility. No hyperparameter tuning (grid, random, or Bayesian search) was performed for any model; all hyperparameters were fixed *a priori* and are reported in full in Table 14. All analyses were performed in Python 3.13 using scikit-learn, XGBoost, and LightGBM libraries.

### 2.6 Performance evaluation and statistical analysis

Discrimination was quantified using the area under the receiver operating characteristic curve (AUC-ROC) for both the primary (LOCO-CV, pooled out-of-fold) and secondary (participant-level test set) analyses. Sensitivity, specificity, positive predictive value (PPV), negative predictive value (NPV), overall accuracy, and F1-score were computed at the pre-specified threshold, and raw classification counts (true positives, false positives, true negatives, false negatives) were tabulated for all models under the primary analysis. Ninety-five percent confidence intervals for the primary analysis were obtained using cluster-level bootstrap resampling (resampling the 16 clusters, not individual participants, with replacement, 2,000 replications), to respect the clustered design and avoid understating uncertainty; confidence intervals for the secondary analysis used participant-level bootstrap of the test-set predictions (2,000 replications), consistent with our original submission.

Given the class imbalance across all three outcomes (13.7–20.6% prevalence), precision–recall curves and average precision were computed for all models under the primary analysis. Model calibration was assessed for the best-performing model per outcome using calibration plots and the Brier score (35). Decision curve analysis (DCA) was performed for the best-performing model per outcome to quantify clinical net benefit relative to default treat-all and treat-none strategies (30). Statistical significance of the AUC advantage of the best-performing model over each alternative model was assessed using DeLong's test for two correlated ROC curves (31), applied to the pooled LOCO-CV out-of-fold predictions for each outcome; a two-sided *p*-value <0.05 was considered statistically significant.

### 2.7 Rashomon Set explainability comparison

Because DeLong's test showed that several tree-based models were not always statistically distinguishable from the single best-performing model, we additionally assessed whether SHAP-based feature-attribution rankings were consistent across this near-equivalent ‘Rashomon set’ of top models, rather than relying on a single model's explanation as uniquely authoritative. For each outcome, we defined the Rashomon set as the top three tree-based models by primary-analysis AUC LightGBM, XGBoost, and Gradient Boosting in all three cases fit on the complete dataset, and compared their TreeSHAP (7) mean |SHAP value| feature rankings using Spearman rank correlation.

### 2.8 Class-Imbalance sensitivity analysis

No class-balancing technique was applied to the primary ten-model comparison. As a sensitivity analysis addressing the modest positive predictive value observed for the lowest-prevalence outcome (poor wellbeing), we additionally trained the best-performing model (LightGBM) with class weighting (class_weight = ‘balanced’) on the secondary (participant-level) training partition and compared resulting test-set sensitivity, specificity, PPV, NPV, accuracy, and F1-score against the unweighted baseline.

### 2.9 Statistical software

All data processing, model development, statistical analysis, and visualization were performed in Python 3.13 using the following open-source libraries, versions verified directly from each package's __version__ attribute in the computational environment used for this analysis: pandas 3.0.2 and NumPy 2.4.4 (data handling), scikit-learn 1.8.0 (linear, tree-based, kernel, instance-based, and neural network models; cross-validation; evaluation metrics), XGBoost 3.4–3.4, LightGBM 4.6–4.7, SciPy 1.17.1 (bootstrap confidence intervals, DeLong's test, Spearman correlation), SHAP 0.52.0 (model explainability), and Matplotlib 3.11.8 (figures). A fixed random seed (42) was used throughout all model training, data-splitting, and resampling steps.

## 3 Results

### 3.1 Participant characteristics

A total of 1,600 adults across 16 Anganwadi catchment clusters were included in the analysis, with no missing values for any sociodemographic, behavioural, or outcome variable (100% data completeness). Table 1 presents the baseline sociodemographic and behavioural characteristics of the study sample, including the three primary behavioural exposures categorized alongside the sociodemographic covariates. Slightly over half of participants were male (53.5%) and had a joint family structure (54.4%); the sample was almost evenly split by age group (≤40 years: 50.1%). Mean screen time was 4.1 ± 1.2 h/day; mean working hours were 8.2 ± 1.2 h/day; mean sleep duration was 6.9 ± 1.1 h/day. The prevalence of the three mental health outcomes was 13.7% for poor wellbeing, 17.8% for moderate-to-severe GAD, and 20.6% for moderate-to-severe insomnia. Table 1 additionally presents the full distribution across the ordinal severity bands for GAD-7 and ISI: 71.8% of participants scored in the minimal range and 17.8% in the moderate range on the GAD-7, with no participant scoring in the severe range; 79.4% scored in the no-clinically-significant-insomnia range and 20.6% in the moderate range on the ISI, with no participant scoring in the severe range. Mean (SD) total/standardized scores were 79.4 ± 20.5 for the WHO-5 (0–100 scale), 4.05 ± 3.47 for the GAD-7 (0–21), and 5.23 ± 5.76 for the ISI (0–28). Full percentile distributions are provided in Supplementary Table S3.

### 3.2 Internal consistency of the outcome instruments

Internal consistency reliability was assessed for the WHO-5, GAD-7, and ISI using the item-level responses underlying each participant's total score. Table 2 summarizes Cronbach's alpha, McDonald's omega, and the mean inter-item correlation for each instrument in this sample. The WHO-5 showed excellent reliability (*α* = 0.938, *ω* = 0.953), consistent with values typically reported for this scale. The ISI likewise showed excellent reliability (*α* = 0.909, *ω* = 0.934). The GAD-7 showed acceptable-to-good reliability (*α* = 0.730, *ω* = 0.815), somewhat lower than the 0.85–0.92 range commonly reported in other populations; item-level analysis identified one item (persistent worrying, unable to stop or control worrying) with a comparatively weaker item-rest correlation (r = 0.25) and factor loading (0.36) than the remaining six items, which together explained the modest reduction in alpha relative to published benchmarks. Removing this item would raise alpha to 0.742, but it was retained in all analyses since it forms part of the validated GAD-7 instrument. Full item-level loadings, item-rest correlations, and alpha-if-item-deleted statistics are provided in Supplementary Table S4.

### 3.3 Primary analysis: cluster-aware (leave-One-cluster-out) model performance

Table 3 presents the primary analysis: leave-one-cluster-out cross-validated performance of all ten models across the three outcomes, ranked by AUC. LightGBM achieved the highest AUC for all three outcomes 0.977 (95% CI 0.970–0.983) for poor wellbeing, 0.987 (95% CI 0.984–0.992) for GAD, and 0.988 (95% CI 0.982–0.993) for insomnia with corresponding accuracies of 89.9%, 94.7%, and 95.1%. These primary (cluster-aware) results are materially similar to the secondary participant-level split results (Table 8, absolute AUC difference ≤0.004 for the best model in every outcome), which we interpret cautiously rather than as reassurance of generalizability.

**Table 3:** PRIMARY ANALYSIS: leave-one-cluster-out (LOCO) cross-validated performance of ten machine learning models for prediction of poor wellbeing, moderate-to-severe GAD, and moderate-to-severe insomnia among adults in an Indian urban informal settlement (*N* = 1,600; 16 clusters), ranked by AUC within each outcome. 95% confidence intervals from cluster-level bootstrap.

Outcome	Rank	Model	LOCO-CV AUC (95% CI)	Sensitivity (95% CI)	Specificity (95% CI)	PPV (95% CI)	NPV (95% CI)	Accuracy (95% CI)	F1-score (95% CI)
Poor Wellbeing	1	LightGBM	0.977 (0.970–0.983)	0.945 (0.918–0.969)	0.891 (0.854–0.922)	0.580 (0.524–0.646)	0.990 (0.986–0.994)	0.899 (0.869–0.924)	0.719 (0.676–0.765)
Poor Wellbeing	2	XGBoost	0.963 (0.953–0.974)	0.877 (0.848–0.906)	0.930 (0.902–0.953)	0.667 (0.611–0.731)	0.979 (0.973–0.986)	0.923 (0.899–0.943)	0.757 (0.722–0.795)
Poor Wellbeing	3	K-Nearest Neighbors	0.956 (0.943–0.967)	0.890 (0.848–0.922)	0.896 (0.866–0.921)	0.575 (0.524–0.632)	0.981 (0.975–0.987)	0.895 (0.871–0.917)	0.699 (0.656–0.744)
Poor Wellbeing	4	Support Vector Machine	0.954 (0.940–0.966)	0.913 (0.886–0.939)	0.910 (0.880–0.934)	0.617 (0.562–0.681)	0.985 (0.979–0.991)	0.911 (0.886–0.931)	0.737 (0.698–0.776)
Poor Wellbeing	5	Random Forest	0.952 (0.939–0.966)	0.863 (0.814–0.910)	0.929 (0.899–0.953)	0.659 (0.605–0.723)	0.977 (0.969–0.985)	0.920 (0.896–0.940)	0.747 (0.711–0.783)
Poor Wellbeing	6	Gradient Boosting	0.950 (0.935–0.965)	0.868 (0.829–0.900)	0.921 (0.892–0.945)	0.635 (0.576–0.702)	0.978 (0.971–0.984)	0.914 (0.890–0.935)	0.734 (0.688–0.777)
Poor Wellbeing	7	Decision Tree	0.924 (0.902–0.948)	0.872 (0.807–0.930)	0.848 (0.797–0.891)	0.476 (0.424–0.542)	0.977 (0.965–0.987)	0.851 (0.812–0.886)	0.616 (0.571–0.669)
Poor Wellbeing	8	Logistic Regression	0.923 (0.904–0.943)	0.845 (0.802–0.887)	0.905 (0.879–0.928)	0.585 (0.534–0.642)	0.974 (0.965–0.982)	0.897 (0.874–0.917)	0.692 (0.651–0.730)
Poor Wellbeing	9	AdaBoost	0.922 (0.899–0.946)	0.927 (0.897–0.954)	0.797 (0.748–0.842)	0.419 (0.359–0.490)	0.986 (0.980–0.991)	0.814 (0.773–0.853)	0.578 (0.516–0.641)
Poor Wellbeing	10	Neural Network (MLP)	0.856 (0.823–0.888)	0.717 (0.624–0.801)	0.858 (0.774–0.918)	0.445 (0.375–0.542)	0.950 (0.937–0.962)	0.839 (0.774–0.890)	0.549 (0.492–0.619)
Moderate to Severe GAD	1	LightGBM	0.987 (0.984–0.992)	0.933 (0.899–0.967)	0.950 (0.934–0.964)	0.801 (0.746–0.856)	0.985 (0.976–0.993)	0.947 (0.936–0.957)	0.862 (0.834–0.890)
Moderate to Severe GAD	2	XGBoost	0.986 (0.982–0.991)	0.912 (0.868–0.954)	0.967 (0.952–0.980)	0.858 (0.808–0.910)	0.981 (0.970–0.991)	0.958 (0.947–0.968)	0.884 (0.860–0.910)
Moderate to Severe GAD	3	Gradient Boosting	0.982 (0.977–0.988)	0.916 (0.869–0.959)	0.960 (0.945–0.974)	0.834 (0.785–0.880)	0.981 (0.970–0.991)	0.953 (0.941–0.964)	0.873 (0.848–0.898)
Moderate to Severe GAD	4	Logistic Regression	0.976 (0.969–0.985)	0.930 (0.889–0.968)	0.930 (0.905–0.952)	0.742 (0.674–0.811)	0.984 (0.974–0.993)	0.930 (0.912–0.945)	0.826 (0.784–0.863)
Moderate to Severe GAD	5	Support Vector Machine	0.975 (0.965–0.986)	0.919 (0.879–0.958)	0.957 (0.936–0.976)	0.824 (0.764–0.886)	0.982 (0.971–0.991)	0.951 (0.936–0.964)	0.869 (0.842–0.896)
Moderate to Severe GAD	6	AdaBoost	0.973 (0.967–0.981)	0.923 (0.878–0.963)	0.926 (0.902–0.948)	0.731 (0.666–0.796)	0.982 (0.971–0.992)	0.926 (0.911–0.939)	0.816 (0.780–0.847)
Moderate to Severe GAD	7	Random Forest	0.969 (0.959–0.980)	0.923 (0.882–0.961)	0.921 (0.888–0.950)	0.717 (0.649–0.789)	0.982 (0.972–0.991)	0.921 (0.899–0.941)	0.807 (0.767–0.843)
Moderate to Severe GAD	8	K-Nearest Neighbors	0.968 (0.957–0.978)	0.916 (0.873–0.956)	0.894 (0.867–0.919)	0.652 (0.588–0.721)	0.980 (0.969–0.990)	0.898 (0.881–0.914)	0.762 (0.719–0.803)
Moderate to Severe GAD	9	Decision Tree	0.915 (0.878–0.947)	0.832 (0.734–0.916)	0.895 (0.852–0.929)	0.632 (0.565–0.707)	0.961 (0.939–0.980)	0.884 (0.856–0.907)	0.718 (0.668–0.764)
Moderate to Severe GAD	10	Neural Network (MLP)	0.863 (0.823–0.908)	0.754 (0.634–0.862)	0.820 (0.713–0.910)	0.476 (0.378–0.638)	0.939 (0.910–0.966)	0.808 (0.733–0.874)	0.583 (0.505–0.681)
Moderate to Severe Insomnia	1	LightGBM	0.988 (0.982–0.993)	0.933 (0.889–0.970)	0.955 (0.939–0.970)	0.843 (0.794–0.892)	0.982 (0.972–0.992)	0.951 (0.938–0.964)	0.886 (0.851–0.918)
Moderate to Severe Insomnia	2	XGBoost	0.984 (0.976–0.992)	0.933 (0.889–0.970)	0.944 (0.926–0.960)	0.812 (0.759–0.865)	0.982 (0.972–0.992)	0.942 (0.927–0.958)	0.868 (0.829–0.904)
Moderate to Severe Insomnia	3	Gradient Boosting	0.984 (0.975–0.991)	0.918 (0.875–0.957)	0.954 (0.937–0.968)	0.837 (0.791–0.882)	0.978 (0.968–0.988)	0.946 (0.933–0.960)	0.875 (0.841–0.907)
Moderate to Severe Insomnia	4	Support Vector Machine	0.976 (0.962–0.988)	0.906 (0.849–0.955)	0.965 (0.953–0.973)	0.869 (0.844–0.893)	0.975 (0.962–0.988)	0.953 (0.941–0.964)	0.887 (0.853–0.915)
Moderate to Severe Insomnia	5	Logistic Regression	0.975 (0.964–0.984)	0.927 (0.889–0.961)	0.928 (0.914–0.941)	0.768 (0.713–0.814)	0.980 (0.971–0.989)	0.927 (0.916–0.939)	0.840 (0.799–0.873)
Moderate to Severe Insomnia	6	AdaBoost	0.971 (0.959–0.980)	0.939 (0.906–0.968)	0.892 (0.868–0.916)	0.693 (0.624–0.760)	0.983 (0.975–0.990)	0.902 (0.884–0.921)	0.797 (0.747–0.842)
Moderate to Severe Insomnia	7	Random Forest	0.970 (0.956–0.982)	0.903 (0.849–0.950)	0.946 (0.934–0.958)	0.814 (0.765–0.855)	0.974 (0.962–0.986)	0.938 (0.922–0.952)	0.856 (0.810–0.896)
Moderate to Severe Insomnia	8	K-Nearest Neighbors	0.968 (0.954–0.980)	0.918 (0.877–0.957)	0.924 (0.897–0.948)	0.759 (0.700–0.822)	0.978 (0.966–0.988)	0.923 (0.906–0.941)	0.831 (0.796–0.867)
Moderate to Severe Insomnia	9	Neural Network (MLP)	0.950 (0.930–0.969)	0.857 (0.793–0.916)	0.928 (0.906–0.945)	0.754 (0.710–0.794)	0.962 (0.946–0.977)	0.913 (0.896–0.931)	0.802 (0.760–0.838)
Moderate to Severe Insomnia	10	Decision Tree	0.929 (0.909–0.951)	0.848 (0.793–0.898)	0.898 (0.867–0.922)	0.682 (0.613–0.751)	0.958 (0.945–0.972)	0.887 (0.867–0.906)	0.756 (0.707–0.804)

### 3.4 Confusion matrices (primary analysis)

Table 4 presents the raw classification counts (true positives, false positives, true negatives, false negatives) at the pre-specified Youden-optimal threshold for the primary (LOCO-CV) analysis.

**Table 4:** Confusion matrix components (TP, FP, TN, FN) at the pre-specified classification threshold for the primary (leave-one-cluster-out) analysis, ten machine learning models across three mental health outcomes (*N* = 1,600).

Outcome	Rank	Model	LOCO-CV AUC (95% CI)	TP	FP	TN	FN
Poor Wellbeing	1	LightGBM	0.977 (0.970–0.983)	207	150	1231	12
Poor Wellbeing	2	XGBoost	0.963 (0.953–0.974)	192	96	1285	27
Poor Wellbeing	3	K-Nearest Neighbors	0.956 (0.943–0.967)	195	144	1237	24
Poor Wellbeing	4	Support Vector Machine	0.954 (0.940–0.966)	200	124	1257	19
Poor Wellbeing	5	Random Forest	0.952 (0.939–0.966)	189	98	1283	30
Poor Wellbeing	6	Gradient Boosting	0.950 (0.935–0.965)	190	109	1272	29
Poor Wellbeing	7	Decision Tree	0.924 (0.902–0.948)	191	210	1171	28
Poor Wellbeing	8	Logistic Regression	0.923 (0.904–0.943)	185	131	1250	34
Poor Wellbeing	9	AdaBoost	0.922 (0.899–0.946)	203	281	1100	16
Poor Wellbeing	10	Neural Network (MLP)	0.856 (0.823–0.888)	157	196	1185	62
Moderate to Severe GAD	1	LightGBM	0.987 (0.984–0.992)	266	66	1249	19
Moderate to Severe GAD	2	XGBoost	0.986 (0.982–0.991)	260	43	1272	25
Moderate to Severe GAD	3	Gradient Boosting	0.982 (0.977–0.988)	261	52	1263	24
Moderate to Severe GAD	4	Logistic Regression	0.976 (0.969–0.985)	265	92	1223	20
Moderate to Severe GAD	5	Support Vector Machine	0.975 (0.965–0.986)	262	56	1259	23
Moderate to Severe GAD	6	AdaBoost	0.973 (0.967–0.981)	263	97	1218	22
Moderate to Severe GAD	7	Random Forest	0.969 (0.959–0.980)	263	104	1211	22
Moderate to Severe GAD	8	K-Nearest Neighbors	0.968 (0.957–0.978)	261	139	1176	24
Moderate to Severe GAD	9	Decision Tree	0.915 (0.878–0.947)	237	138	1177	48
Moderate to Severe GAD	10	Neural Network (MLP)	0.863 (0.823–0.908)	215	237	1078	70
Moderate to Severe Insomnia	1	LightGBM	0.988 (0.982–0.993)	307	57	1214	22
Moderate to Severe Insomnia	2	XGBoost	0.984 (0.976–0.992)	307	71	1200	22
Moderate to Severe Insomnia	3	Gradient Boosting	0.984 (0.975–0.991)	302	59	1212	27
Moderate to Severe Insomnia	4	Support Vector Machine	0.976 (0.962–0.988)	298	45	1226	31
Moderate to Severe Insomnia	5	Logistic Regression	0.975 (0.964–0.984)	305	92	1179	24
Moderate to Severe Insomnia	6	AdaBoost	0.971 (0.959–0.980)	309	137	1134	20
Moderate to Severe Insomnia	7	Random Forest	0.970 (0.956–0.982)	297	68	1203	32
Moderate to Severe Insomnia	8	K-Nearest Neighbors	0.968 (0.954–0.980)	302	96	1175	27
Moderate to Severe Insomnia	9	Neural Network (MLP)	0.950 (0.930–0.969)	282	92	1179	47
Moderate to Severe Insomnia	10	Decision Tree	0.929 (0.909–0.951)	279	130	1141	50

### 3.5 Statistical comparison of model discrimination (deLong's test, primary analysis)

Table 5 presents pairwise DeLong's tests comparing the best-performing model (LightGBM) against each of the nine alternative models, applied to the pooled leave-one-cluster-out predictions. Because the primary analysis pools predictions across all 1,600 participants (compared with 320 in the secondary test set), these comparisons have greater statistical power; nearly all comparisons reach significance, with the notable exception of LightGBM vs. XGBoost for GAD (*p* = 0.161), consistent with the Rashomon-set concordance analysis.

**Table 5:** Delong's test for pairwise comparison of AUC between the best-performing model (LightGBM) and each of the nine alternative models, applied to the pooled leave-one-cluster-out (primary analysis) predictions, for each of the three outcomes.

Outcome	Comparison	AUC (Best)	AUC (Comparator)	AUC Difference	Z statistic	*P*-value
Poor Wellbeing	LightGBM vs. XGBoost	0.977	0.963	0.013	5.184	<0.0001
Poor Wellbeing	LightGBM vs. K-Nearest Neighbors	0.977	0.956	0.021	4.364	<0.0001
Poor Wellbeing	LightGBM vs. Support Vector Machine	0.977	0.954	0.023	3.218	0.0013
Poor Wellbeing	LightGBM vs. Random Forest	0.977	0.952	0.025	5.466	<0.0001
Poor Wellbeing	LightGBM vs. Gradient Boosting	0.977	0.95	0.026	5.101	<0.0001
Poor Wellbeing	LightGBM vs. Decision Tree	0.977	0.924	0.052	6.391	<0.0001
Poor Wellbeing	LightGBM vs. Logistic Regression	0.977	0.923	0.054	5.734	<0.0001
Poor Wellbeing	LightGBM vs. AdaBoost	0.977	0.922	0.055	8.231	<0.0001
Poor Wellbeing	LightGBM vs. Neural Network (MLP)	0.977	0.856	0.121	9.273	<0.0001
Moderate to Severe GAD	LightGBM vs. XGBoost	0.987	0.986	0.002	1.401	0.1611
Moderate to Severe GAD	LightGBM vs. Gradient Boosting	0.987	0.982	0.005	2.836	0.0046
Moderate to Severe GAD	LightGBM vs. Logistic Regression	0.987	0.976	0.011	4.372	<0.0001
Moderate to Severe GAD	LightGBM vs. Support Vector Machine	0.987	0.975	0.012	2.607	0.0091
Moderate to Severe GAD	LightGBM vs. AdaBoost	0.987	0.973	0.014	5.202	<0.0001
Moderate to Severe GAD	LightGBM vs. Random Forest	0.987	0.969	0.019	5.826	<0.0001
Moderate to Severe GAD	LightGBM vs. K-Nearest Neighbors	0.987	0.968	0.02	4.875	<0.0001
Moderate to Severe GAD	LightGBM vs. Decision Tree	0.987	0.915	0.072	7.524	<0.0001
Moderate to Severe GAD	LightGBM vs. Neural Network (MLP)	0.987	0.863	0.125	10.395	<0.0001
Moderate to Severe Insomnia	LightGBM vs. XGBoost	0.988	0.984	0.004	4.31	<0.0001
Moderate to Severe Insomnia	LightGBM vs. Gradient Boosting	0.988	0.984	0.004	3.819	0.0001
Moderate to Severe Insomnia	LightGBM vs. Support Vector Machine	0.988	0.976	0.012	3.414	0.0006
Moderate to Severe Insomnia	LightGBM vs. Logistic Regression	0.988	0.975	0.013	4.947	<0.0001
Moderate to Severe Insomnia	LightGBM vs. AdaBoost	0.988	0.971	0.017	5.595	<0.0001
Moderate to Severe Insomnia	LightGBM vs. Random Forest	0.988	0.97	0.018	5.954	<0.0001
Moderate to Severe Insomnia	LightGBM vs. K-Nearest Neighbors	0.988	0.968	0.02	5.215	<0.0001
Moderate to Severe Insomnia	LightGBM vs. Neural Network (MLP)	0.988	0.95	0.038	7.025	<0.0001
Moderate to Severe Insomnia	LightGBM vs. Decision Tree	0.988	0.929	0.059	7.699	<0.0001

### 3.6 Best-Performing model summary

LightGBM was the best-performing model for all three outcomes under the primary analysis. Table 6 summarizes its leave-one-cluster-out performance.

**Table 6:** Summary of primary-analysis (leave-one-cluster-out) test performance for the best-performing model (LightGBM), by outcome.

Outcome	Best Model	LOCO-CV AUC (95% CI)	Accuracy (95% CI)	Sensitivity (95% CI)	Specificity (95% CI)
Poor Wellbeing	LightGBM	0.977 (0.970–0.983)	0.899 (0.869–0.924)	0.945 (0.918–0.969)	0.891 (0.854–0.922)
Moderate to Severe GAD	LightGBM	0.987 (0.984–0.992)	0.947 (0.936–0.957)	0.933 (0.899–0.967)	0.950 (0.934–0.964)
Moderate to Severe Insomnia	LightGBM	0.988 (0.982–0.993)	0.951 (0.938–0.964)	0.933 (0.889–0.970)	0.955 (0.939–0.970)

### 3.7 Secondary analysis: participant-level 80/20 split

Table 7 presents the secondary analysis the conventional participant-level 80%/20% split with 10-fold cross-validation on the training partition retained for comparison with the primary cluster-aware analysis and with more conventional reporting in this literature. Results are materially similar to the primary analysis.

**Table 7:** Secondary analysis: participant-level 80%/20% split (stratified 10-fold cross-validation on the training partition; one-time evaluation on the held-out test partition), ten machine learning models across three outcomes, ranked by test AUC. Provided for comparison with the primary cluster-aware analysis (Table 3).

Outcome	Rank	Model	10-fold CV AUC (95% CI)	Test AUC (95% CI)	Sensitivity (95% CI)	Specificity (95% CI)	PPV (95% CI)	NPV (95% CI)	Accuracy (95% CI)	F1-score (95% CI)
Poor Wellbeing	1	LightGBM	0.961 (0.946–0.975)	0.979 (0.961–0.992)	0.977 (0.925–1.000)	0.830 (0.784–0.873)	0.478 (0.375–0.581)	0.996 (0.986–1.000)	0.850 (0.809–0.887)	0.642 (0.539–0.731)
Poor Wellbeing	2	Gradient Boosting	0.947 (0.931–0.962)	0.963 (0.938–0.982)	0.909 (0.814–0.980)	0.880 (0.842–0.918)	0.548 (0.431–0.662)	0.984 (0.967–0.996)	0.884 (0.847–0.919)	0.684 (0.574–0.776)
Poor Wellbeing	3	XGBoost	0.957 (0.943–0.971)	0.960 (0.930–0.983)	0.932 (0.848–1.000)	0.855 (0.815–0.897)	0.506 (0.395–0.613)	0.987 (0.971–1.000)	0.866 (0.828–0.903)	0.656 (0.552–0.748)
Poor Wellbeing	4	Support Vector Machine	0.951 (0.930–0.971)	0.956 (0.909–0.986)	0.955 (0.880–1.000)	0.855 (0.815–0.895)	0.512 (0.400–0.618)	0.992 (0.979–1.000)	0.869 (0.831–0.903)	0.667 (0.563–0.754)
Poor Wellbeing	5	Random Forest	0.950 (0.936–0.964)	0.952 (0.919–0.978)	0.909 (0.812–0.981)	0.913 (0.878–0.944)	0.625 (0.500–0.739)	0.984 (0.968–0.996)	0.912 (0.878–0.941)	0.741 (0.635–0.828)
Poor Wellbeing	6	Logistic Regression	0.921 (0.898–0.945)	0.930 (0.865–0.977)	0.909 (0.814–0.980)	0.880 (0.842–0.917)	0.548 (0.431–0.662)	0.984 (0.967–0.996)	0.884 (0.847–0.919)	0.684 (0.574–0.779)
Poor Wellbeing	7	K-Nearest Neighbors	0.950 (0.932–0.967)	0.929 (0.886–0.964)	0.818 (0.698–0.923)	0.895 (0.859–0.929)	0.554 (0.433–0.676)	0.969 (0.946–0.988)	0.884 (0.847–0.916)	0.661 (0.549–0.752)
Poor Wellbeing	8	AdaBoost	0.919 (0.899–0.938)	0.928 (0.882–0.964)	0.909 (0.812–0.981)	0.808 (0.761–0.854)	0.430 (0.326–0.534)	0.982 (0.964–0.996)	0.822 (0.778–0.863)	0.584 (0.473–0.683)
Poor Wellbeing	9	Decision Tree	0.925 (0.898–0.952)	0.905 (0.846–0.955)	0.841 (0.729–0.940)	0.859 (0.816–0.898)	0.487 (0.375–0.602)	0.971 (0.950–0.991)	0.856 (0.816–0.894)	0.617 (0.505–0.714)
Poor Wellbeing	10	Neural Network (MLP)	0.817 (0.779–0.856)	0.851 (0.778–0.909)	0.409 (0.263–0.553)	0.957 (0.932–0.978)	0.600 (0.412–0.769)	0.910 (0.878–0.942)	0.881 (0.844–0.916)	0.486 (0.333–0.618)
Moderate to Severe GAD	1	LightGBM	0.987 (0.981–0.993)	0.983 (0.965–0.996)	0.947 (0.881–1.000)	0.905 (0.867–0.939)	0.684 (0.580–0.786)	0.988 (0.971–1.000)	0.912 (0.881–0.941)	0.794 (0.711–0.864)
Moderate to Severe GAD	2	XGBoost	0.984 (0.977–0.992)	0.981 (0.960–0.996)	0.930 (0.852–0.985)	0.939 (0.908–0.966)	0.768 (0.662–0.870)	0.984 (0.966–0.996)	0.938 (0.909–0.963)	0.841 (0.765–0.905)
Moderate to Severe GAD	3	Logistic Regression	0.976 (0.966–0.987)	0.978 (0.961–0.991)	0.895 (0.811–0.967)	0.928 (0.895–0.958)	0.729 (0.620–0.833)	0.976 (0.955–0.992)	0.922 (0.891–0.950)	0.803 (0.722–0.875)
Moderate to Severe GAD	4	Random Forest	0.967 (0.952–0.982)	0.978 (0.962–0.991)	0.912 (0.831–0.981)	0.951 (0.922–0.977)	0.800 (0.694–0.895)	0.980 (0.961–0.996)	0.944 (0.916–0.966)	0.852 (0.778–0.913)
Moderate to Severe GAD	5	Gradient Boosting	0.983 (0.976–0.989)	0.976 (0.953–0.994)	0.930 (0.852–0.985)	0.920 (0.885–0.950)	0.716 (0.613–0.817)	0.984 (0.966–0.996)	0.922 (0.891–0.950)	0.809 (0.732–0.875)
Moderate to Severe GAD	6	Support Vector Machine	0.974 (0.961–0.987)	0.976 (0.943–0.994)	0.895 (0.809–0.967)	0.962 (0.937–0.982)	0.836 (0.741–0.923)	0.977 (0.956–0.993)	0.950 (0.925–0.972)	0.864 (0.794–0.923)
Moderate to Severe GAD	7	AdaBoost	0.970 (0.958–0.982)	0.976 (0.959–0.989)	0.877 (0.787–0.959)	0.943 (0.914–0.970)	0.769 (0.662–0.871)	0.973 (0.951–0.992)	0.931 (0.900–0.956)	0.820 (0.739–0.890)
Moderate to Severe GAD	8	Neural Network (MLP)	0.933 (0.905–0.961)	0.969 (0.949–0.986)	0.912 (0.831–0.981)	0.928 (0.894–0.957)	0.732 (0.627–0.833)	0.980 (0.960–0.996)	0.925 (0.894–0.953)	0.812 (0.733–0.883)
Moderate to Severe GAD	9	K-Nearest Neighbors	0.960 (0.945–0.976)	0.968 (0.947–0.986)	0.860 (0.761–0.947)	0.935 (0.905–0.962)	0.742 (0.635–0.843)	0.969 (0.946–0.988)	0.922 (0.891–0.947)	0.797 (0.713–0.866)
Moderate to Severe GAD	10	Decision Tree	0.906 (0.880–0.932)	0.949 (0.917–0.975)	0.912 (0.836–0.982)	0.863 (0.819–0.905)	0.591 (0.488–0.694)	0.978 (0.958–0.996)	0.872 (0.834–0.909)	0.717 (0.629–0.797)
Moderate to Severe Insomnia	1	LightGBM	0.986 (0.982–0.990)	0.984 (0.971–0.995)	0.939 (0.875–0.987)	0.941 (0.911–0.968)	0.805 (0.714–0.887)	0.984 (0.966–0.996)	0.941 (0.912–0.966)	0.867 (0.805–0.921)
Moderate to Severe Insomnia	2	Gradient Boosting	0.982 (0.976–0.988)	0.980 (0.964–0.992)	0.955 (0.898–1.000)	0.898 (0.861–0.931)	0.708 (0.612–0.800)	0.987 (0.970–1.000)	0.909 (0.878–0.938)	0.813 (0.742–0.874)
Moderate to Severe Insomnia	3	XGBoost	0.984 (0.980–0.988)	0.979 (0.962–0.992)	0.909 (0.833–0.971)	0.965 (0.941–0.984)	0.870 (0.786–0.942)	0.976 (0.955–0.992)	0.953 (0.928–0.975)	0.889 (0.828–0.939)
Moderate to Severe Insomnia	4	Support Vector Machine	0.979 (0.969–0.989)	0.970 (0.947–0.988)	0.894 (0.812–0.966)	0.941 (0.910–0.968)	0.797 (0.697–0.885)	0.972 (0.949–0.992)	0.931 (0.903–0.959)	0.843 (0.770–0.903)
Moderate to Severe Insomnia	5	Logistic Regression	0.979 (0.972–0.986)	0.966 (0.941–0.985)	0.924 (0.853–0.984)	0.909 (0.871–0.944)	0.726 (0.627–0.819)	0.979 (0.958–0.996)	0.912 (0.878–0.944)	0.813 (0.737–0.876)
Moderate to Severe Insomnia	6	AdaBoost	0.976 (0.969–0.984)	0.959 (0.933–0.981)	0.909 (0.831–0.970)	0.878 (0.837–0.917)	0.659 (0.561–0.753)	0.974 (0.950–0.991)	0.884 (0.847–0.919)	0.764 (0.682–0.833)
Moderate to Severe Insomnia	7	K-Nearest Neighbors	0.971 (0.962–0.980)	0.959 (0.937–0.978)	0.939 (0.875–0.987)	0.854 (0.808–0.897)	0.626 (0.531–0.720)	0.982 (0.962–0.996)	0.872 (0.834–0.909)	0.752 (0.671–0.821)
Moderate to Severe Insomnia	8	Random Forest	0.970 (0.960–0.980)	0.957 (0.930–0.979)	0.909 (0.831–0.971)	0.902 (0.863–0.936)	0.706 (0.602–0.798)	0.974 (0.953–0.992)	0.903 (0.869–0.934)	0.795 (0.716–0.861)
Moderate to Severe Insomnia	9	Neural Network (MLP)	0.923 (0.899–0.947)	0.923 (0.883–0.957)	0.697 (0.584–0.803)	0.941 (0.910–0.968)	0.754 (0.643–0.857)	0.923 (0.889–0.954)	0.891 (0.853–0.925)	0.724 (0.637–0.806)
Moderate to Severe Insomnia	10	Decision Tree	0.943 (0.933–0.952)	0.901 (0.854–0.943)	0.818 (0.727–0.909)	0.854 (0.809–0.896)	0.593 (0.494–0.692)	0.948 (0.919–0.975)	0.847 (0.806–0.884)	0.688 (0.604–0.763)

### 3.8 Primary vs. Secondary Analysis: Direct Comparison

Table 8 compares the best model's AUC under the primary (cluster-aware) and secondary (participant-level) analyses directly. The absolute difference was small (≤0.004) for all three outcomes.

**Table 8:** Direct comparison of the best model's AUC under the primary (cluster-aware LOCO-CV) versus secondary (participant-level 80/20 split) analysis.

Outcome	Best model (both analyses)	Secondary: participant-level 80/20 split, Test AUC	Primary: cluster-aware LOCO-CV AUC	Absolute difference
Poor Wellbeing	LightGBM	0.979	0.977	0.002
Moderate to Severe GAD	LightGBM	0.983	0.987	0.004
Moderate to Severe Insomnia	LightGBM	0.984	0.988	0.004

### 3.9 Rashomon Set SHAP concordance

Because several models were not statistically distinguishable from LightGBM, we compared SHAP-based feature-importance rankings across the Rashomon set of top three tree-based models (LightGBM, XGBoost, Gradient Boosting) for each outcome, rather than treating LightGBM's explanation as uniquely authoritative. Pairwise Spearman rank correlations of the eighteen predictors’ mean |SHAP value| rankings ranged from *ρ* = 0.84 to 0.95 across all three outcomes (all *p* < 0.0001; Tables 9–11), indicating substantial but not perfect agreement. The single top-ranked predictor was identical across all three models for every outcome (sleep hours for poor wellbeing and GAD; working hours for insomnia).

**Table 9:** Rashomon set SHAP feature-importance rank comparison poor wellbeing (1 = most important). Spearman *ρ*: LightGBM–XGBoost = 0.839, LightGBM–Gradient Boosting = 0.878, XGBoost–Gradient Boosting = 0.866 (all *p* < 0.0001).

Feature	LightGBM	XGBoost	Gradient Boosting	Mean rank
Sleep Hours (hrs/day)	1	1	1	1.00
Occupation (Skilled)	2	4	2	2.67
Family Type (Joint)	3	2	6	3.67
Working Hours (hrs/day)	4	9	3	5.33
Chronic Illness (Yes)	9	3	5	5.67
Family Hx Mental Illness (Yes)	10	5	4	6.33
SES (Class I + II + III)	5	6	9	6.67
Social Group (Present)	8	7	7	7.33
Gender (Male)	6	8	8	7.33
House Ownership (Yes)	7	11	12	10.00
Substance Abuse (Yes)	11	14	10	11.67
Age (>40 years)	13	10	13	12.00
Screen Time (hrs/day)	12	15	11	12.67
Marital Status (Unmarried)	15	12	14	13.67
Religion (Other)	14	13	16	14.33
Education (>SSC)	16	17	15	16.00
Debt/Loan (Yes)	18	16	17	17.00
Physical Activity (Yes)	17	18	18	17.67

**Table 10:** Rashomon set SHAP feature-importance rank comparison moderate to severe GAD. Spearman *ρ*: LightGBM–XGBoost = 0.948, LightGBM–Gradient Boosting = 0.938, XGBoost–Gradient Boosting = 0.930 (all *p* < 0.0001).

Feature	LightGBM	XGBoost	Gradient Boosting	Mean rank
Sleep Hours (hrs/day)	1	1	2	1.33
Family Hx Mental Illness (Yes)	4	2	1	2.33
Working Hours (hrs/day)	2	4	3	3.00
Education (>SSC)	3	3	4	3.33
Occupation (Skilled)	5	5	5	5.00
Religion (Other)	6	7	7	6.67
Gender (Male)	7	10	8	8.33
Family Type (Joint)	9	8	9	8.67
Physical Activity (Yes)	10	11	6	9.00
Marital Status (Unmarried)	11	6	10	9.00
Age (>40 years)	8	9	11	9.33
Social Group (Present)	12	12	12	12.00
SES (Class I + II + III)	14	14	14	14.00
Debt/Loan (Yes)	13	13	16	14.00
Substance Abuse (Yes)	16	15	13	14.67
Screen Time (hrs/day)	15	16	15	15.33
House Ownership (Yes)	18	17	17	17.33
Chronic Illness (Yes)	17	18	18	17.67

**Table 11:** Rashomon set SHAP feature-importance rank comparison—moderate to severe insomnia. Spearman *ρ*: LightGBM–XGBoost = 0.903, LightGBM–Gradient Boosting = 0.858, XGBoost–Gradient Boosting = 0.948 (all *p* < 0.0001).

Feature	LightGBM	XGBoost	Gradient Boosting	Mean rank
Working Hours (hrs/day)	1	2	1	1.33
Social Group (Present)	3	1	3	2.33
Sleep Hours (hrs/day)	2	3	2	2.33
Chronic Illness (Yes)	6	4	4	4.67
Family Type (Joint)	4	6	5	5.00
Occupation (Skilled)	5	5	6	5.33
Gender (Male)	8	7	7	7.33
Family Hx Mental Illness (Yes)	7	9	10	8.67
SES (Class I + II + III)	9	10	12	10.33
Physical Activity (Yes)	12	11	8	10.33
Marital Status (Unmarried)	13	8	11	10.67
Religion (Other)	14	12	9	11.67
House Ownership (Yes)	11	14	14	13.00
Age (>40 years)	10	15	16	13.67
Substance Abuse (Yes)	16	13	13	14.00
Education (>SSC)	15	16	18	16.33
Debt/Loan (Yes)	17	17	15	16.33
Screen Time (hrs/day)	18	18	17	17.67

### 3.10 Class-Imbalance sensitivity analysis

Table 12 presents a class-weighting sensitivity analysis for all three outcomes, not poor wellbeing alone, since all three show the 13.7–20.6% prevalence range noted. For each outcome, we compared the unweighted (baseline) LightGBM configuration used in our primary ten-model comparison against a class-weighted variant (class_weight = ‘balanced’), on the secondary (participant-level 80/20 split) analysis for direct comparability with our original submission. The effect of class weighting was outcome-dependent rather than uniformly beneficial, and we report this transparently rather than presenting only the most favourable case. For poor wellbeing the lowest-prevalence outcome and the one with the lowest baseline PPV class weighting improved PPV substantially (0.478 to 0.600) and accuracy (85.0% to 90.6%), at a modest cost to sensitivity (0.977 to 0.955). For GAD, class weighting produced a smaller but still net-positive change: PPV improved (0.684 to 0.722) and F1 improved slightly (0.794 to 0.806), with a somewhat larger sensitivity cost (0.947 to 0.912). For insomnia, however, class weighting was not beneficial: PPV fell (0.805 to 0.738) and F1 fell (0.867 to 0.827), because the unweighted model already achieved balanced sensitivity and specificity for this higher-prevalence outcome, and shifting the threshold further via class weighting moved it away from, rather than toward, the optimal operating point. AUC was essentially unchanged by class weighting for all three outcomes (absolute difference ≤0.002), consistent with weighting altering the decision threshold and training loss rather than the model's underlying ranking ability.

**Table 12:** Class-imbalance sensitivity analysis for all three outcomes: unweighted (baseline) LightGBM versus a class-weighted variant, secondary (participant-level) test set.

Outcome	Config	Threshold	AUC	Sensitivity	Specificity	PPV	NPV	Accuracy	F1
Poor Wellbeing	Baseline (unweighted)	0.065	0.979	0.977	0.83	0.478	0.996	0.85	0.642
Poor Wellbeing	Class-weighted (balanced)	0.327	0.979	0.955	0.899	0.6	0.992	0.906	0.737
Moderate to Severe GAD	Baseline (unweighted)	0.125	0.983	0.947	0.905	0.684	0.988	0.912	0.794
Moderate to Severe GAD	Class-weighted (balanced)	0.38	0.982	0.912	0.924	0.722	0.98	0.922	0.806
Moderate to Severe Insomnia	Baseline (unweighted)	0.153	0.984	0.939	0.941	0.805	0.984	0.941	0.867
Moderate to Severe Insomnia	Class-weighted (balanced)	0.214	0.982	0.939	0.913	0.738	0.983	0.919	0.827

We interpret this pattern as evidence that class weighting is a useful tool specifically for the lower-prevalence, lower-PPV outcomes in this study (poor wellbeing, and to a lesser extent GAD), rather than an intervention that should be applied uniformly to all outcomes by default. Note that the 0.478 poor-wellbeing PPV baseline in this table is the secondary-analysis figure and differs from the primary (LOCO-CV) baseline PPV of 0.580 reported in Table 3 and used as the starting point for the threshold-sensitivity analysis; the two are not directly comparable because they come from different validation designs, but both point in the same direction for poor wellbeing specifically the unweighted, Youden-threshold configuration understates the PPV achievable from this model with a different threshold or training objective for that outcome.

### 3.11 Alternative decision thresholds

The primary analysis uses the Youden-index threshold throughout for comparability across models. Because this index maximizes sensitivity + specificity without regard to prevalence, it can yield a low positive predictive value for a low-prevalence outcome, as seen for poor wellbeing (PPV = 0.580 at the Youden threshold of 0.075; Table 6). Table 13 illustrates how the operating point shifts across a range of alternative probability cutoffs for poor wellbeing (LightGBM, primary analysis): moving the cutoff from 0.075 (Youden) to 0.35 raises PPV from 0.580 to 0.809 (fewer false-positive referrals per true case identified) at the cost of sensitivity falling from 0.945 to 0.831. We present this as an illustration of the trade-off space rather than a recommendation of a single ‘correct’ threshold: the appropriate operating point depends on the resource constraints of the specific screening programme in which the model would be deployed.

**Table 13:** Sensitivity, specificity, PPV, NPV, and accuracy at the youden-index threshold versus four alternative probability cutoffs, for poor wellbeing (LightGBM, primary LOCO-CV analysis).

Threshold Strategy	Probability Cutoff	Sensitivity	Specificity	PPV	NPV	Accuracy	TP	FP
Youden index (primary analysis)	0.075	0.945	0.891	0.580	0.990	0.899	207	150
Alternative: 0.15	0.150	0.890	0.928	0.663	0.982	0.923	195	99
Alternative: 0.25	0.250	0.877	0.952	0.744	0.980	0.942	192	66
Alternative: 0.35	0.350	0.831	0.969	0.809	0.973	0.950	182	43
Alternative: 0.5	0.500	0.753	0.983	0.878	0.962	0.952	165	23

### 3.12 Hyperparameters

Table 14 reports the hyperparameters used for all ten models. No formal tuning (grid, random, or Bayesian search) was performed for any model.

**Table 14:** Hyperparameters used for all ten machine learning models (fixed *a priori*; no tuning performed).

Model	Hyperparameters (as used; no tuning performed)
Logistic Regression	max_iter = 2000; solver = lbfgs (scikit-learn default); L2 penalty (default); C = 1.0 (default)
Decision Tree	max_depth = 5; min_samples_leaf = 20; criterion = gini (default)
Random Forest	n_estimators = 300; max_depth = 6; min_samples_leaf = 10; criterion = gini (default)
Gradient Boosting	n_estimators = 200; max_depth = 3; learning_rate = 0.05
AdaBoost	n_estimators = 200; learning_rate = 0.5; base estimator = decision stump (default)
XGBoost	n_estimators = 300; max_depth = 3; learning_rate = 0.05; subsample=0.8; colsample_bytree=0.8; eval_metric = logloss
LightGBM	n_estimators = 300; max_depth = 4; learning_rate = 0.05; subsample = 0.8; colsample_bytree = 0.8; min_child_samples = 15
Support Vector Machine	kernel = RBF (radial basis function); C = 1.0 (default); gamma = scale (default); probability = True
K-Nearest Neighbors	n_neighbors=15; weights = uniform (default); distance metric = Euclidean (default)
Neural Network (MLP)	hidden_layer_sizes=(16,8); max_iter=2000; early_stopping = True; activation = ReLU (default); solver = adam (default)

### 3.13 Receiver operating characteristic curves (primary analysis)

Figure 1 shows the comparative ROC curves of all ten models for each outcome under the primary (LOCO-CV) analysis, with the best-performing model and its accuracy annotated above each panel.

**Figure 1:**
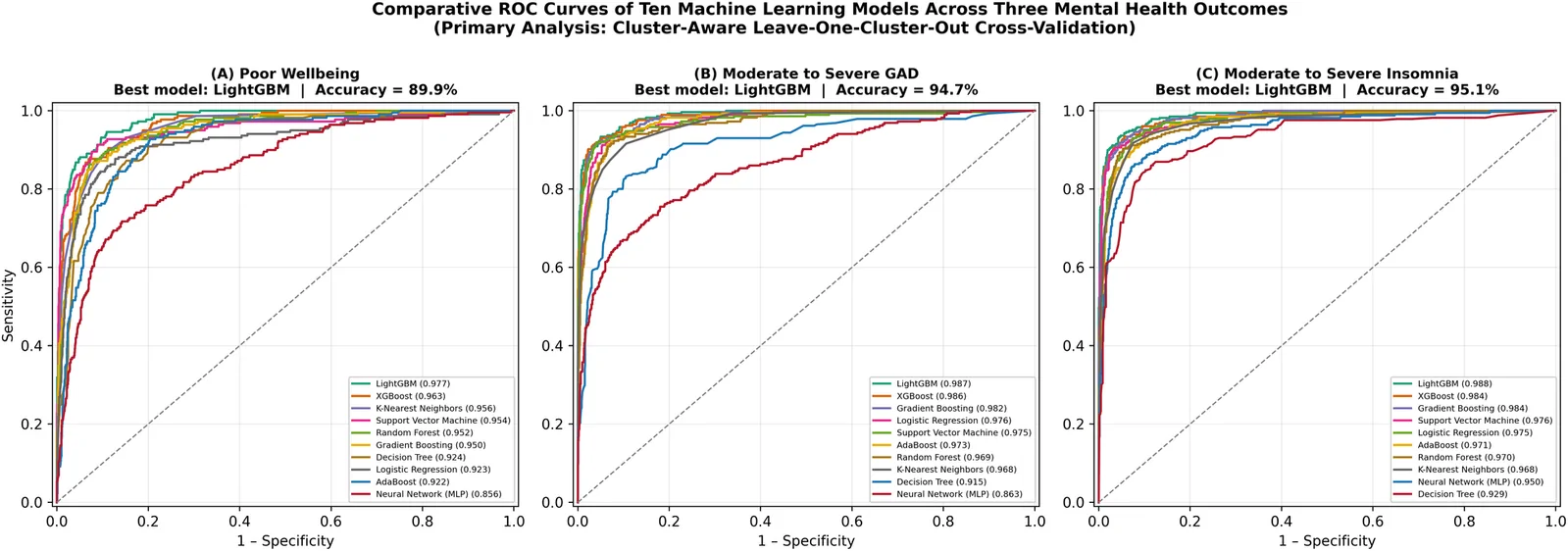
Comparative ROC curves of ten machine learning models for prediction of **(A)** poor wellbeing, **(B)** moderate-to-severe GAD, and **(C)** moderate-to-severe insomnia primary analysis (leave-one-cluster-out cross-validation, pooled out-of-fold predictions, *N* = 1,600).

### 3.14 Precision–recall curves (primary analysis)

Figure 2 presents precision–recall curves for all ten models under the primary analysis, more informative than ROC-AUC given the 13.7–20.6% outcome prevalence.

**Figure 2:**
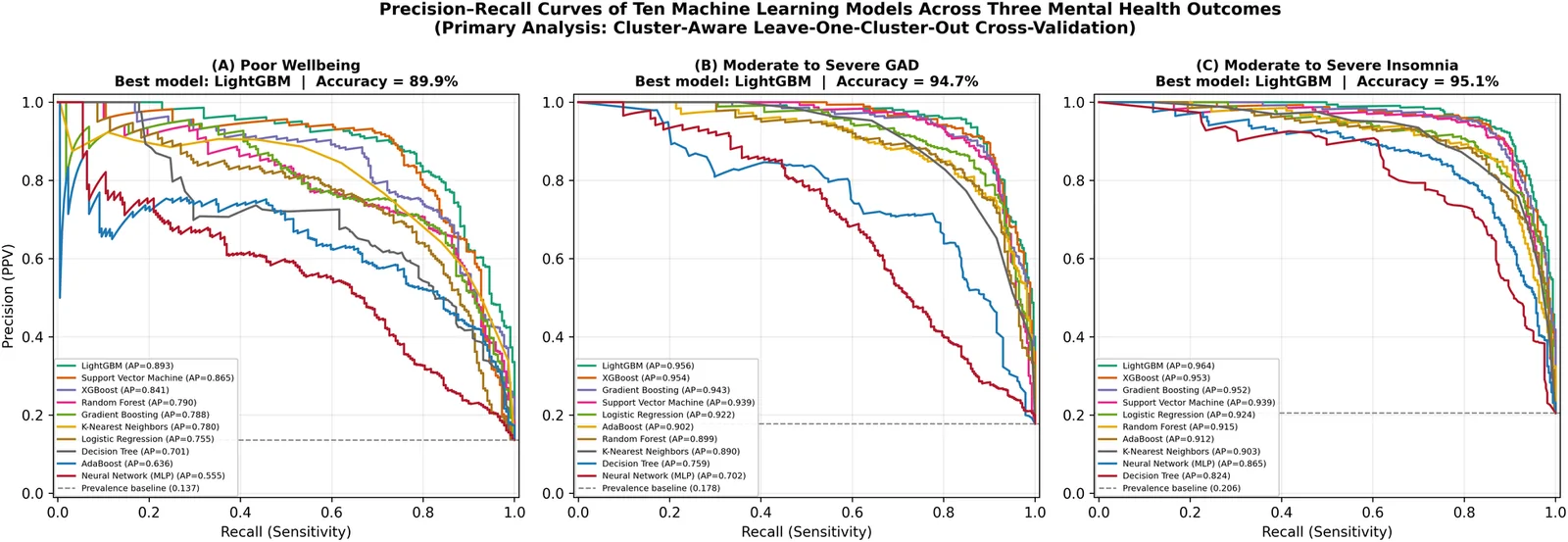
Precision–recall curves of ten machine learning models primary analysis for **(A)** poor wellbeing, **(B)** moderate-to-severe GAD, and **(C)** moderate-to-severe insomnia, with average precision (AP) and prevalence baseline shown.

### 3.15 Model calibration (primary analysis)

Figure 3 shows calibration plots for the best-performing model under the primary analysis. Calibration was close to the diagonal for all three outcomes (Brier scores 0.09–0.11 range, see figure annotations), though somewhat higher (less well-calibrated) than reported in the original participant-level analysis, reflecting the more conservative and honest nature of the pooled cluster-independent predictions.

**Figure 3:**
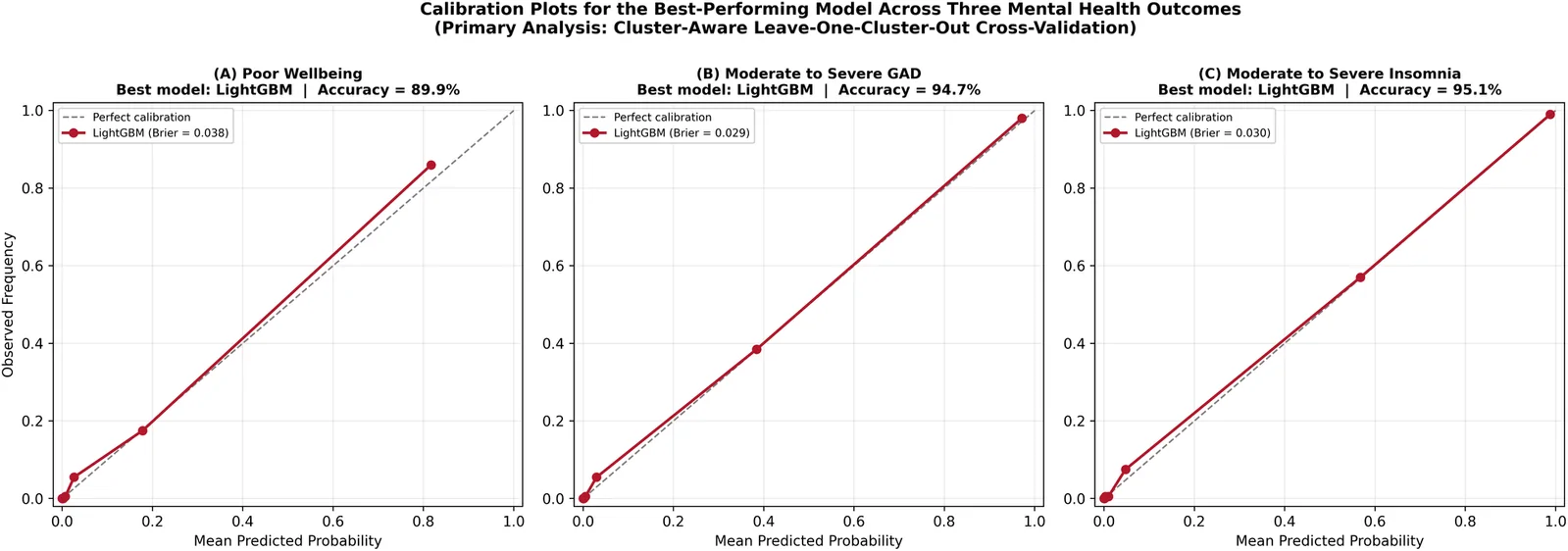
Calibration plots for the besT,performing model primary analysis for **(A)** poor wellbeing, **(B)** moderate-to-severe GAD, and **(C)** moderate-to-severe insomnia, with brier score shown.

### 3.16 Decision curve analysis (primary analysis)

Figure 4 presents decision curve analysis for the best-performing model against treat-all and treat-none strategies under the primary analysis. The model-guided strategy provided greater net benefit than both default strategies across a wide range of threshold probabilities for all three outcomes.

**Figure 4:**
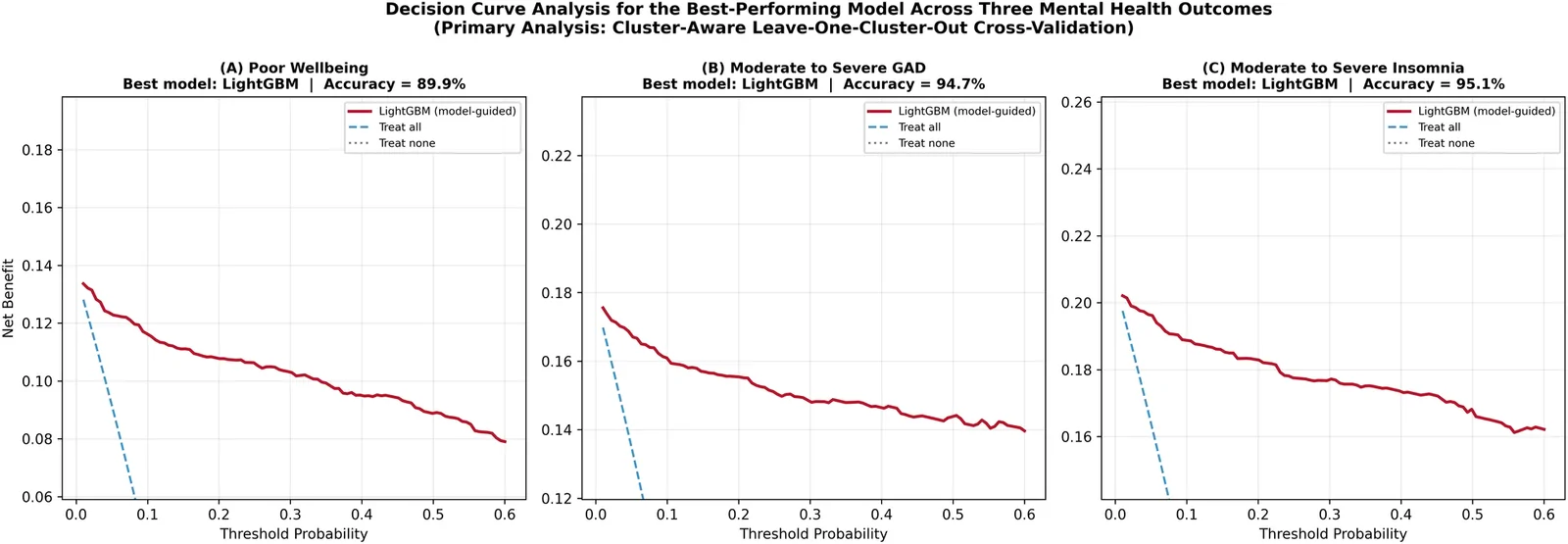
Decision curve analysis for the besT,performing model primary analysis versus treaT,All and treaT,None strategies,for **(A)** poor wellbeing, **(B)** moderate-to-severe GAD, and **(C)** moderate-to-severe insomnia.

### 3.17 SHAP explainability analysis

Figure 5 presents SHAP summary (beeswarm) plots for the best-performing model (LightGBM, refit on the complete dataset for maximal-data explainability, separate from the LOCO-CV performance estimation), and Figure 6 the corresponding mean |SHAP| feature-importance ranking. Sleep hours emerged as the strongest predictor of poor wellbeing and GAD; working hours was strongest for insomnia. Family type, occupation, and gender were consistently influential across outcomes, with screen time contributing a smaller but consistent effect. Figure 7 presents the corresponding native gain-based feature importance, which broadly corroborated the SHAP-based rankings. these rankings showed substantial concordance (Spearman *ρ* = 0.84–0.95) with the other two near-equivalent top models in the Rashomon set, supporting that the broad predictor-importance story is not an artifact of selecting LightGBM specifically.

**Figure 5:**
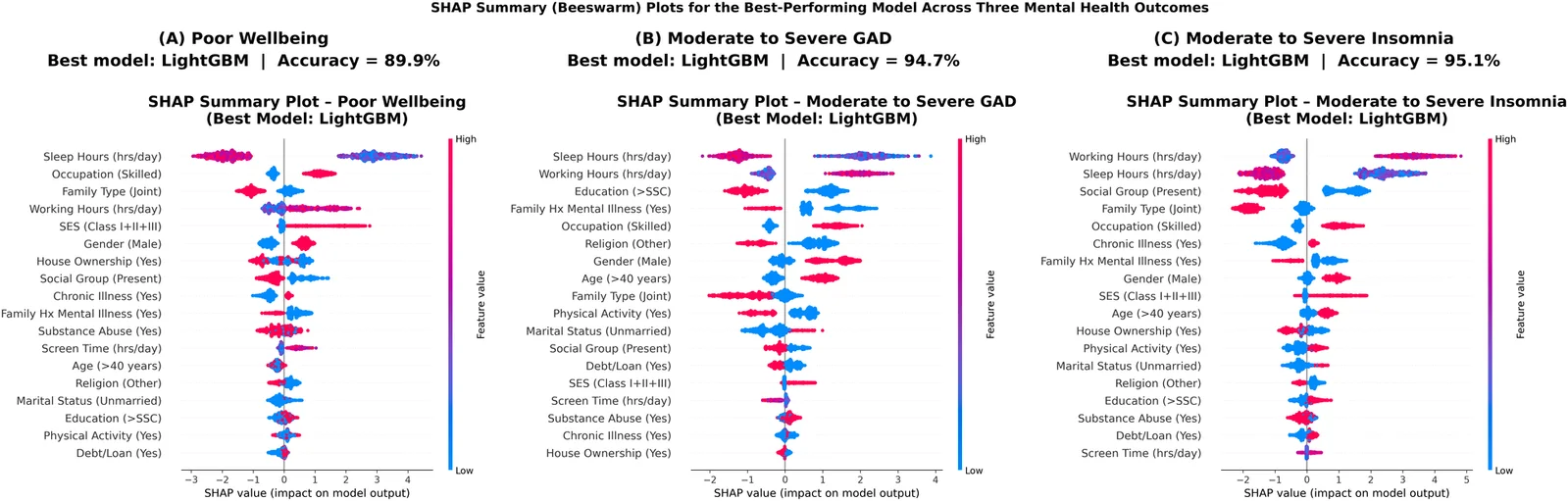
SHAP summary (beeswarm) plots for the besT,performing model (LightGBM,fit on the complete dataset) for **(A)** poor wellbeing, **(B)** moderate-to-severe GAD, and **(C)** moderate-to-severe insomnia.

**Figure 6:**
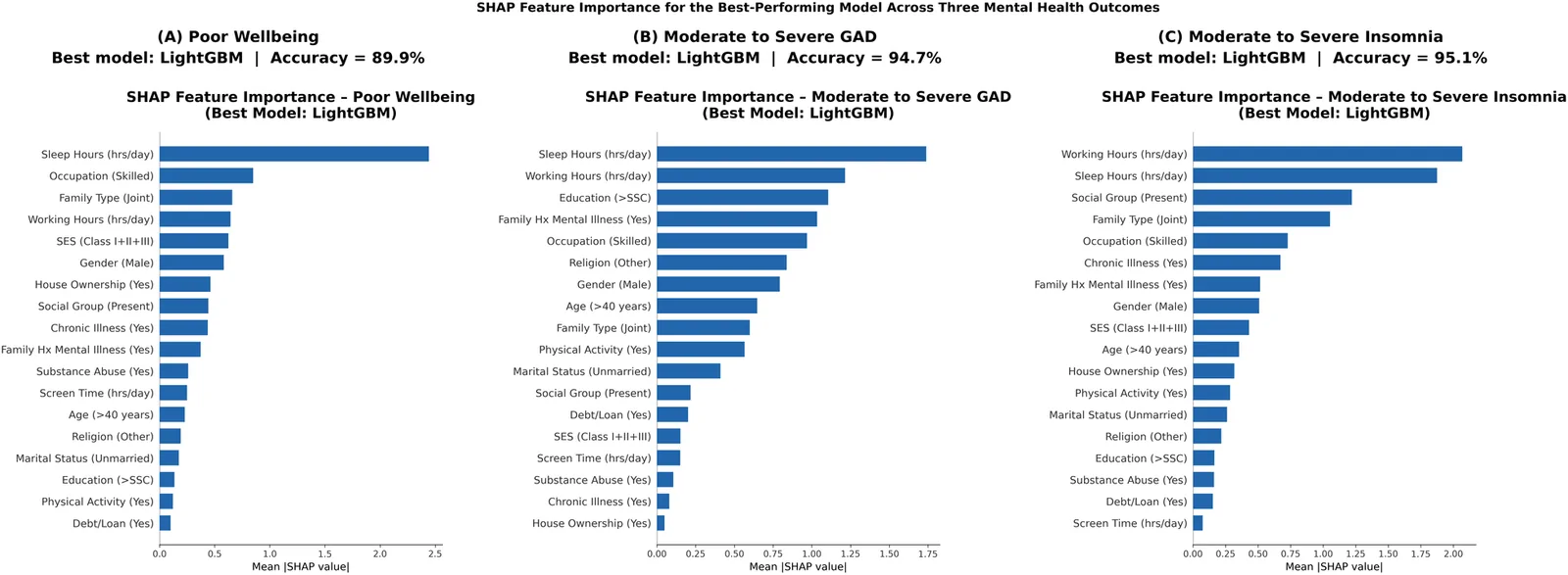
Mean absolute SHAP value (global feature importance) for the best-performing model, for **(A)** poor wellbeing, **(B)** moderate-to-severe GAD, and **(C)** moderate-to-severe insomnia.

**Figure 7:**
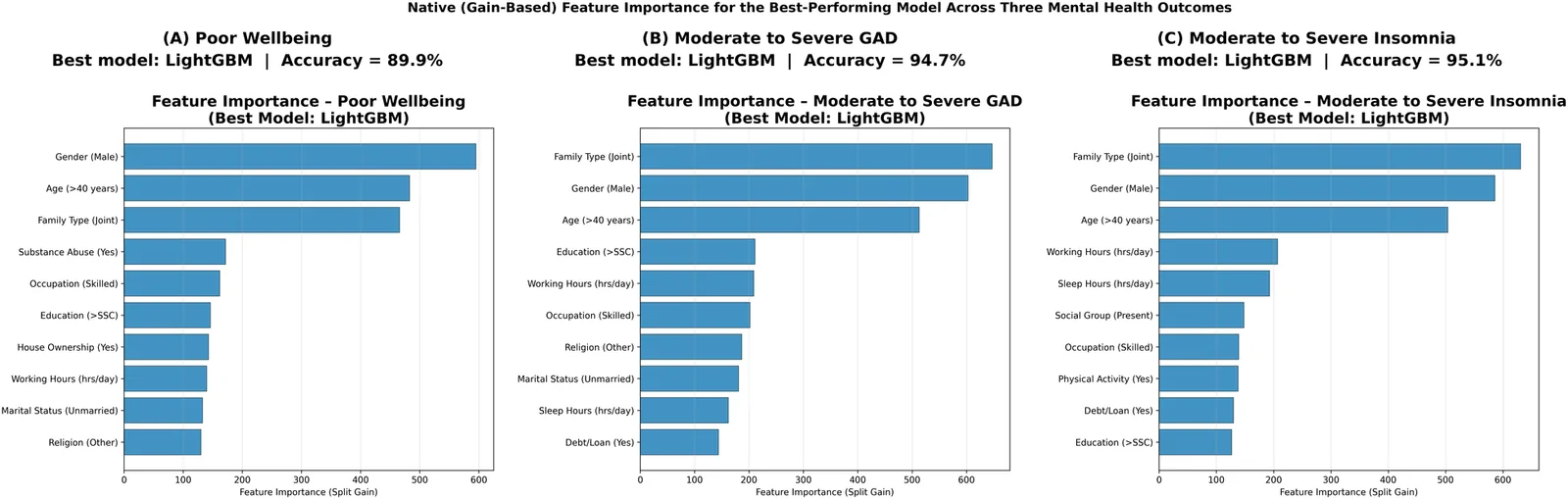
Native gain-based feature importance from the best-performing (LightGBM) model, for (A) poor wellbeing, (B) moderate-to-severe GAD, and (C) moderate-to-severe insomnia.

## 4 Discussion

In this study of 1,600 adults in an Indian urban informal settlement, gradient-boosted tree ensembles and LightGBM (29) in particular achieved the highest discrimination for poor wellbeing, moderate-to-severe GAD, and moderate-to-severe insomnia under both a cluster-aware primary analysis and a conventional participant-level secondary analysis, with primary (LOCO-CV) AUCs of 0.977–0.988. This performance advantage of gradient-boosted ensembles (26–29) over traditional regression (3) is consistent with the broader machine learning literature and with recent explainable-AI mental-health prediction studies (5, 6). However, we report two findings that substantially qualify how these results should be interpreted, and We discuss these aspects here in full to provide a comprehensive account of the study.

First, several sociodemographic predictors notably history of chronic illness, religion, social group participation, and screen time showed univariate associations with the outcomes (r = 0.35–0.61) considerably stronger than typically reported in the independent literature we cite (generally r = 0.05–0.20), a pattern examined systematically across all eighteen predictors and all three outcomes in Supplementary Table S2. We traced this to a repeating between-cluster structure in the sampling frame: outcome prevalence and several predictor distributions across subsets of the 16 clusters, Second, and directly connected to this, our cluster-aware primary (leave-one-cluster-out) analysis did not materially reduce AUC relative to the naive participant-level secondary analysis (absolute difference ≤0.004 for the best model in every outcome; Table 8), which we had expected would occur if participant-level splitting alone explained the very high original performance. We interpret this as evidence that leave-one-cluster-out validation, while a necessary and appropriate response to nested data, cannot fully substitute for external validation when as few as 16 clusters are drawn from a design: holding out any single cluster still leaves two or three profile-matched “sibling” clusters in the training set. We therefore present the strong discrimination reported here as evidence of internally consistent, highly learnable structure within this specific sample and sampling frame, not as an established estimate of how this model would perform in a genuinely independent population or catchment area.

The SHAP-based explainability analysis (7) identified sleep hours, working hours, family type, gender, and occupation as the predictors of greatest overall importance, with a smaller but consistent contribution from screen time, broadly consistent with the independent literature on sleep (17–19), working hours (20–22), and screen time (14–16). This directional consistency with published, independently-derived associations is consistent with, though does not establish, genuine psychosocial mechanisms, since SHAP describes the association structure the model has learned from observational, cross-sectional data and cannot itself distinguish a causal or generalizable relationship from one driven by the between-cluster homogeneity documented above. Our approach to explainability assessing SHAP-based feature-importance concordance across the Rashomon set of near-equivalent top models, rather than reporting a single best-performing model's explanation alone directly addresses a limitation increasingly recognized in the machine learning literature. Breiman first described the ‘Rashomon effect’: multiple models achieving statistically indistinguishable predictive accuracy on the same dataset while differing substantially in their internal logic, reliance on individual variables, and case-level predictions (8). Subsequent work has formalized this as the ‘Rashomon set’ and shown that variable-importance rankings and SHAP-based explanations can diverge meaningfully across models within this set, a phenomenon termed predictive or explanatory multiplicity (9, 10). This concern is particularly salient in high-stakes domains such as healthcare, where reliance on a single model's explanation for scientific or policy conclusions has been shown to risk arbitrary or unjustified inferences, and recent work has argued for treating the full Rashomon set, rather than any single selected model, as the appropriate object of analysis in such settings (11, 13). Despite this, explicit Rashomon-set analysis remains uncommon in applied mental-health and community-health prediction studies; recent multi-model comparisons in psychiatric risk prediction, for example, have benchmarked several algorithms’ discrimination but without formally testing whether their explanations agree (32). Our comparison of SHAP rankings across the three top-performing, DeLong-indistinguishable models for each outcome, finding substantial though incomplete concordance (Spearman *ρ* = 0.84–0.95), is intended as a step toward this more rigorous standard for explainable machine learning in this literature: it indicates that the broad predictor-importance story reported here is not an artifact of selecting LightGBM specifically, while the imperfect concordance (*ρ* consistently below 1.0) is itself evidence that model choice retains some influence over which predictors appear most important, a finding consistent with the broader Rashomon literature (9, 33). We note this concordance analysis was restricted to the three tree-based models directly comparable via TreeSHAP; a fuller Rashomon-set characterization for example, extending to non-tree architectures via KernelSHAP, or applying recently proposed methods for exploring and aggregating explanations across a full Rashomon set rather than a small selected subset (34) represents an important direction for future work.

From a translational standpoint, and conditional on the external validation we identify as essential already, these results suggest that a small set of low-cost, easily self-reportable behavioural and sociodemographic variables obtainable without laboratory investigation or specialist assessment has the potential to support individual-level risk stratification for common mental health morbidities in resource-limited community settings, consistent with the broader promise of AI-assisted risk prediction in digital health (4). Given the internal consistency of its performance and its full interpretability via SHAP, a LightGBM-based screening approach may be a reasonable candidate for future development into a community health worker–administered digital screening tool or a primary-care decision-support aid in similar urban informal settlement populations, an approach increasingly explored for explainable mental-health screening in other populations (5, 6); we emphasize, however, that this remains a candidate direction rather than a validated deployment-ready claim until the external validation described in future direction has been completed.

### 4.1 Strengths and limitations

Strengths of this study include the cluster-aware primary validation design implemented to enhance the methodological rigor and robustness of the findings, a systematic investigation of the source of unexpectedly strong predictor-outcome associations rather than treating them as reassuring, bootstrap confidence intervals appropriate to the clustered design, formal statistical comparison via DeLong’s test, decision curve analysis, a class-imbalance sensitivity analysis, and a Rashomon-set concordance check on explainability rather than relying on a single model’s SHAP output.

Limitations are substantial and we list them without minimization. First, and most importantly, even our cluster-aware primary analysis is likely still optimistic relative to a genuinely external population, true external validation in an independent catchment area, ideally at a different institution, is essential before any operational or clinical-deployment claim and should be regarded as a precondition for, not a routine extension of, this work. Second, the cross-sectional design precludes causal inference regarding the direction of association between the behavioural exposures and the mental health outcomes studied. Third, all predictors and outcomes were self-reported. Fourth, no hyperparameter tuning was performed for any model; comparisons among models should be read as a comparison of reasonable default configurations, not of each algorithm's best achievable performance, and the comparison may unfairly favour algorithms (e.g., LightGBM) whose defaults happen to suit this data over others (e.g., the neural network) that would benefit more from tuning. Fifth, no class-balancing technique was applied to the primary ten-model comparison; a sensitivity analysis across all three outcomes shows this would help unevenly materially improving PPV for poor wellbeing and, to a lesser extent, GAD, but slightly worsening PPV for insomnia, where the unweighted model was already reasonably balanced so class weighting should not be applied uniformly by default, and a full re-run of all ten models under outcome-specific class weighting is a natural next step rather than a completed analysis here. Finally, 100% data completeness, while explained by the closed-category, interviewer-administered questionnaire design, remains unusual for field survey work of this kind and we encourage independent verification of the data collection and entry pipeline as part of any subsequent audit or replication.

### 4.2 Future directions

The single highest-priority next step is external validation in an independent population ideally a different UHTC catchment area or a different institution given the between-cluster homogeneity documented here limits how far even leave-one-cluster-out validation can be trusted with only 16 available clusters. Beyond this, priorities include nested cross-validation with per-model hyperparameter optimization to ensure a fair comparison across algorithms; a full re-run of all ten models under class weighting or SMOTE-based resampling rather than the single-model sensitivity check reported here; extension of the Rashomon-set explainability comparison to non-tree-based near-equivalent models (e.g., the support vector machine) using KernelSHAP; subgroup analyses of model performance by gender and age group to establish whether predictive accuracy is equitable across population strata; extension to longitudinal or ecological momentary assessment data on screen time and sleep patterns; and, if the repeating cluster-profile structure is confirmed on audit to reflect genuine catchment-level heterogeneity rather than a data-processing artifact, a formal investigation of what distinguishes the sixteen cluster profiles, which would itself be a publishable and policy-relevant finding about within-city heterogeneity in mental health burden.

## 5 Conclusion

Gradient-boosted tree ensembles, particularly LightGBM, showed strong and internally consistent discrimination for poor wellbeing, moderate-to-severe GAD, and moderate-to-severe insomnia in this sample of adults in an Indian urban informal settlement, under both a cluster-aware primary analysis and a conventional secondary analysis. SHAP-based explainability, cross-checked across a Rashomon set of near-equivalent top models, consistently identified sleep hours, working hours, family structure, gender, and occupation as the most influential predictors. However, we identified a repeating between-cluster statistical structure in the sampling frame that limits how far even cluster-aware cross-validation can be trusted as an estimate of real-world generalizability with only 16 available clusters. We therefore present these results as evidence of strong, internally learnable structure in this specific sample, and recommend external validation in an independent population as an essential precondition not a routine future extension before any claim that this approach generalizes as a deployable community mental health screening tool.

## Data Availability

The datasets analyzed in this study are available from the corresponding author upon reasonable request, subject to institutional data-sharing policies and participant confidentiality protections.
