# Supplementary material for: Machine learning based multimodel prediction of poor wellbeing, generalized anxiety disorder, and insomnia among adults in Indian urban informal settlements: toward explainable digital mental health screening
Source: Front Digit Health. 2026 Sep 30;8:1935205. doi: 10.3389/fdgth.2026.1935205 (PMC13661956; doi:10.3389/fdgth.2026.1935205)
Supplement: Supplementary file 1 [file Table1.pdf]

Supplementary Tables

Supplementary Table S1. Cluster-Level Statistics Documenting the Repeating Between-Cluster Profile Pattern

Outcome prevalence and selected predictor means for each of the 16 sampling clusters (100 participants per cluster), documenting the five recurring statistical profiles discussed in Section 4.1 of the main manuscript. Referenced in Section 2.4.

Clusters	N	Poor Wellbeing prevalence	GAD prevalence	Insomnia prevalence	Religion coded mean	History Chronic Illness coded mean	Social Group coded mean	Screen time mean	sleep hours mean	Debt loan coded mean	working hours mean
1	100	0.09	0.15	0.11	0.29	0.06	0.75	3.486	7.372	0.19	8.035
2	100	0.15	0.17	0.25	0.40	0.19	0.55	3.764	7.172	0.40	7.993
3	100	0.18	0.24	0.25	0.25	0.20	0.61	3.926	7.028	0.33	7.913
4	100	0.07	0.07	0.13	0.07	0.11	0.53	3.80	7.281	0.43	8.058
5	100	0.21	0.27	0.32	0.03	0.26	0.52	3.975	6.983	0.41	7.953
6	100	0.10	0.16	0.13	0.00	0.07	0.77	3.741	6.968	0.19	8.032
7	100	0.13	0.18	0.24	0.04	0.17	0.58	4.037	6.773	0.40	8.135
8	100	0.14	0.25	0.26	0.01	0.21	0.59	4.121	6.927	0.33	8.176
9	100	0.10	0.06	0.12	0.08	0.13	0.54	4.193	6.957	0.43	8.272
10	100	0.19	0.24	0.30	0.35	0.22	0.48	4.328	6.676	0.41	8.355
11	100	0.11	0.12	0.14	0.21	0.08	0.71	4.294	6.816	0.19	8.293
12	100	0.17	0.16	0.26	0.68	0.16	0.57	4.389	6.680	0.40	8.418
13	100	0.18	0.22	0.23	0.44	0.22	0.63	4.448	6.652	0.33	8.358
14	100	0.07	0.13	0.12	0.12	0.13	0.53	4.201	6.911	0.43	8.439
15	100	0.21	0.25	0.31	0.60	0.24	0.50	4.836	6.530	0.41	8.391
16	100	0.09	0.18	0.12	0.10	0.07	0.77	4.298	6.717	0.19	8.277

Supplementary Table S2. Univariate (Single-Predictor) Association Check

Univariate AUC and discriminative power of each of the 18 candidate predictors against each of the three outcomes, used to screen for possible target leakage or collinearity prior to model development. Referenced in Section 4.

Outcome	Predictor	Univariate AUC	Discriminative power (max(AUC,1-AUC))
GAD	sleep hours	0.194	0.806
GAD	Screen time	0.805	0.805
GAD	Social Group coded	0.219	0.781
GAD	Religion coded	0.76	0.76
GAD	History Chronic Illness coded	0.748	0.748
GAD	House ownership coded	0.348	0.652
GAD	Debt loan coded	0.636	0.636
GAD	Type of family coded	0.391	0.609
GAD	working hours	0.584	0.584
GAD	Age coded	0.563	0.563
GAD	Any Substance abuse coded	0.54	0.54
GAD	Gender coded	0.467	0.533
GAD	Occupation coded	0.471	0.529
GAD	Family History of Mental Health coded	0.528	0.528

Outcome	Predictor	Univariate AUC	Discriminative power (max(AUC,1-AUC))
GAD	Marital Status coded	0.525	0.525
GAD	Socioeconomic Status coded	0.481	0.519
GAD	Education coded	0.491	0.509
GAD	Physical Activity coded	0.501	0.501
Insomnia	sleep hours	0.184	0.816
Insomnia	Social Group coded	0.217	0.783
Insomnia	History Chronic Illness coded	0.776	0.776
Insomnia	Screen time	0.771	0.771
Insomnia	Religion coded	0.731	0.731
Insomnia	Debt loan coded	0.66	0.66
Insomnia	House ownership coded	0.383	0.617
Insomnia	Any Substance abuse coded	0.585	0.585
Insomnia	working hours	0.572	0.572
Insomnia	Socioeconomic Status coded	0.444	0.556
Insomnia	Age coded	0.543	0.543
Insomnia	Education coded	0.472	0.528
Insomnia	Type of family coded	0.473	0.527
Insomnia	Physical Activity coded	0.477	0.523
Insomnia	Family History of Mental Health coded	0.521	0.521
Insomnia	Marital Status coded	0.519	0.519
Insomnia	Occupation coded	0.511	0.511
Insomnia	Gender coded	0.492	0.508
Poor Wellbeing	Social Group coded	0.202	0.798
Poor Wellbeing	sleep hours	0.21	0.79
Poor Wellbeing	Screen time	0.771	0.771
Poor Wellbeing	Religion coded	0.753	0.753
Poor Wellbeing	History Chronic Illness coded	0.718	0.718
Poor Wellbeing	Debt loan coded	0.662	0.662
Poor Wellbeing	House ownership coded	0.41	0.59
Poor Wellbeing	Any Substance abuse coded	0.581	0.581
Poor Wellbeing	Type of family coded	0.431	0.569
Poor Wellbeing	Family History of Mental Health coded	0.554	0.554
Poor Wellbeing	Occupation coded	0.448	0.552
Poor Wellbeing	Marital Status coded	0.539	0.539
Poor Wellbeing	Education coded	0.533	0.533
Poor Wellbeing	Physical Activity coded	0.528	0.528
Poor Wellbeing	working hours	0.528	0.528
Poor Wellbeing	Age coded	0.525	0.525
Poor Wellbeing	Socioeconomic Status coded	0.519	0.519
Poor Wellbeing	Gender coded	0.515	0.515

Supplementary Table S3. Descriptive Statistics for the Three Total/Standardized Outcome Scores

Distributional summary of the WHO-5, GAD-7, and ISI total/standardized scores prior to dichotomization (N = 1,600), including percentiles not reported in the main text.

Score	N	Mean	SD	Min	25%	50%	75%	Max
WHO-5 (0-100 scale)	1600	79.39	20.51	8	76	88	92	100
GAD-7 (0-21)	1600	8.05	2.97	3	4	5	7	19
ISI (0-28)	1600	11.45	4.76	3	5	8	10	24

Supplementary Table S4. Item-Level Reliability Detail

Factor loading, item-rest correlation, and alpha-if-item-deleted for each of the 19 items across the three instruments (5 WHO-5 items, 7 GAD-7 items, 7 ISI items; N = 1,600). Referenced in Section 3.2 of the main manuscript.

Instrument	Item	Factor loading	Item-rest correlation	Alpha if item dropped
WHO-5	WHO1	0.895	0.833	0.925
WHO-5	WHO2	0.914	0.861	0.919
WHO-5	WHO3	0.902	0.843	0.923
WHO-5	WHO4	0.885	0.818	0.927
WHO-5	WHO5	0.886	0.82	0.927
GAD-7	Anxity1	0.707	0.528	0.678
GAD-7	Anxity2	0.658	0.482	0.689
GAD-7	Anxity3	0.364	0.247	0.742
GAD-7	Anxity4	0.625	0.453	0.7
GAD-7	Anxity5	0.661	0.479	0.691
GAD-7	Anxity6	0.697	0.504	0.686
GAD-7	Anxity7	0.614	0.441	0.701
ISI	Sleep1	0.861	0.809	0.888
ISI	Sleep2	0.712	0.623	0.907
ISI	Sleep3	0.754	0.658	0.903
ISI	Sleep4	0.87	0.8	0.896
ISI	Sleep5	0.855	0.782	0.89
ISI	Sleep6	0.844	0.792	0.89
ISI	Sleep7	0.829	0.75	0.894
